# Editorial

**Published:** 1898-04

**Authors:** 


### Editorial.

Dr. Hugh Hagan died of cerebral hemorrhage on Tuesday morning, March 22, at the age of 34 years. Dr. Hagan was a native of Richmond, Virginia. He was graduated from the College of Physicians and Surgeons, New York, with the class of 1889. Immediately after graduation, he removed to Atlanta. He subsequently spent some time in Europe, and on his return to Atlanta resumed his practice, confining it largely to diseases of the mind and nervous system.

Blessed with a cheerful disposition, united with great cordiality of manner, there were very few men in the city who could boast of a larger circle of friends and admirers than Dr. Hagan. As a physician he was clear, decisive, resolute and enthusiastic in his performance of duty. He gained and held the confidence of his patients, and enjoyed the respect and esteem of his professional confreres and the community at large. The recollection of his robust personality, his hearty friendliness, his helpfulness in counsel and his unfailing kindness to all in distress and sickness, will remain forever enshrined in the hearts of his friends. He leaves a wife and two sons to mourn his loss.

---

There are in the United States very few physicians, except the unmarried, the recent graduate, and the restless stick-at-nothing, who will be disposed to volunteer their services at the first outbreak of hostilities with Spain.

War to the doctor is something, to characterize it no stronger, extremely inconvenient. The majority of them, save the elect few who were born with the silver spoon, or of opulent heredity, or have shown great skill in the selection of a father-in-law, are dependent upon the work of their hands and brains for a livelihood and have but little stored away against the time when the evil day cometh.

War to them means desertion of a family poorly equipped to survive in the absence of the bread-winner, dispersion of practice, and after the wrench of parting is over, exposure to disease, hardship and hard work. The surgeons in the late war who were shot down in the performance of duty or fell victims to the ravages of disease which projected its dark shadow over the armies, numbered among the hundreds and bear witness to the fact that instead of being non-combatants, they were confronted by a double menace. It is hardly reasonable to suppose that the sword of war with Spain could hew down to the heart of this great country and make it necessary for every able-bodied man in it to go forth and fight for its preservation. Long before this happened, every doctor in the land would have cheerfully given his services regardless of home ties, for the profession of medicine is not calculated to make poltroons of its devotees. The United States expects every man to do his duty.

---

### COLLEGE COMMENCEMENTS.

The fortieth annual commencement of the Atlanta Medical College took place at the Grand Opera House, March 28, 1898. There were forty-six graduates. The honors were awarded to the following: First honor, Dr. Rios Zertuchi, of Mexico; second, Dr. J. Jones, of South Carolina; third, Dr. J. H. Johns, of South Carolina. The following graduates received honorable mention: Leroy Napier, Ga.; W. H. Johnson, Ga.; J. M. Smith, Ga.; A. J. Moony, Ga.

---

The Southern Medical College held its nineteeth annual commencement on March 30. There were twenty-seven graduates. The honors were awarded to the following gentlemen: First honor, Louis Sage Hardin, S. C.; second, W. A. Tillie; the third was awarded between Aubrey Harper and C. A. Smith, of Ga., Joseph Goodwin, Tenn., J. Weddington, Ga., V. Lagerson, Me., and George Turner, Ga., received honorable mention.

---

The Southern section of the American Laryngological, Rhinological and Otological Society convened in Atlanta, March 28. The list of papers presented was large and interesting. A reçeption was tendered the visiting members by the chairman, Dr. A. W. Calhoun.

---

The Association of Surgeons of the Central Railroad of Georgia, will meet at Cumberland Island, at the same time as the State Association.

